# Wnt/β-catenin signalling in ovarian cancer: Insights into its hyperactivation and function in tumorigenesis

**DOI:** 10.1186/s13048-019-0596-z

**Published:** 2019-12-11

**Authors:** Vu Hong Loan Nguyen, Rebecca Hough, Stefanie Bernaudo, Chun Peng

**Affiliations:** 1 Department of Biology, York University, Toronto, Ontario Canada; 2 Centre for Research on Biomolecular Interactions, York University, Toronto, Ontario Canada

**Keywords:** Ovarian cancer, Wnt/β-catenin signalling, microRNAs, cancer stem cells, metastasis, tumor angiogenesis

## Abstract

Epithelial ovarian cancer (EOC) is the deadliest female malignancy. The Wnt/β-catenin pathway plays critical roles in regulating embryonic development and physiological processes. This pathway is tightly regulated to ensure its proper activity. In the absence of Wnt ligands, β-catenin is degraded by a destruction complex. When the pathway is stimulated by a Wnt ligand, β-catenin dissociates from the destruction complex and translocates into the nucleus where it interacts with TCF/LEF transcription factors to regulate target gene expression. Aberrant activation of this pathway, which leads to the hyperactivity of β-catenin, has been reported in ovarian cancer. Specifically, mutations of *CTNNB1*, *AXIN, or APC,* have been observed in the endometrioid and mucinous subtypes of EOC. In addition, upregulation of the ligands, abnormal activation of the receptors or intracellular mediators, disruption of the β-catenin destruction complex, inhibition of the association of β-catenin/E-cadherin on the cell membrane, and aberrant promotion of the β-catenin/TCF transcriptional activity, have all been reported in EOC, especially in the high grade serous subtype. Furthermore, several non-coding RNAs have been shown to regulate EOC development, in part, through the modulation of Wnt/β-catenin signalling. The Wnt/β-catenin pathway has been reported to promote cancer stem cell self-renewal, metastasis, and chemoresistance in all subtypes of EOC. Emerging evidence also suggests that the pathway induces ovarian tumor angiogenesis and immune evasion. Taken together, these studies demonstrate that the Wnt/β-catenin pathway plays critical roles in EOC development and is a strong candidate for the development of targeted therapies.

## Introduction

Ovarian cancer is the most lethal gynecological malignancy and is ranked as the fifth leading cause of cancer deaths in females [1]. It is estimated that there are 22,530 new cases with a mortality rate of approximately 13,980 deaths in the United States in 2019 [1]. Ovarian cancers are grouped into three categories based on the cell type of origin: epithelial, stromal, and germ cell cancer [2]. Among them, epithelial ovarian cancer (EOC) accounts for 90-95% of ovarian malignancies. EOC is further grouped into five histological subtypes: high-grade serous carcinomas (HGSC, 70%-74%), endometrioid carcinomas (EC, 7-24%), clear cell carcinomas (CCC, 10%- 26%), low-grade serous carcinomas (LGSC, 3%-5%), and mucinous carcinomas (MC, 2%-6%) [3]. The poor survival rate of ovarian cancer patients is mainly due to the lack of screening methods at the early stages and the lack of effective treatments for advanced stages of the disease [4]. The standard chemotherapy for EOC patients is a combination of a platinum product, such as cisplatin or carboplatin, with a taxane, such as paclitaxel or docetaxel [3]. However, many patients develop resistance to these therapies and relapse [5, 6]. Recent research has introduced several therapeutic agents that target specific cancer-driven factors to inhibit ovarian cancer development. For example, bevacizumab, an antibody against vascular endothelial growth factor (VEGF)-A, has been approved by the FDA to be used in combination with carboplatin and paclitaxel [7]. Moreover, several Poly (ADP-Ribose) Polymerase (PARP) inhibitors have been approved for the treatment of recurrent BRCA-mutated EOC [8].

The Wnt/β-catenin pathway regulates cell proliferation, polarity, survival, and stem cell fate in embryonic and adult tissue homeostasis [9]. The pathway is tightly regulated to ensure its proper activity. It is well documented that aberrant Wnt signalling is associated with the development of several pathologies, including cancer [10, 11]. Accumulating evidence shows that the Wnt/β-catenin pathway regulates many key aspects of cancer development, including maintaining cancer stem cells (CSCs); promoting metastasis, cancer cell survival, and chemoresistance [12, 13]; suppressing the immune response within the tumor microenvironment [14, 15]; and enhancing tumor angiogenesis [16]. The role of the Wnt/β-catenin pathway in CSC self-renewal, metastasis, and chemoresistance has been reported in all subtypes of EOC [12, 17]. Recent studies suggest that this pathway is also involved in ovarian tumor angiogenesis [18] and immune evasion [19]. In addition, mutations that lead to the hyperactivity of β-catenin, are commonly observed in the EC subtype [20, 21]. In this review, we will summarize current knowledge of the Wnt/β-catenin signalling cascade, mutations and dysregulation in this pathway that result in the hyperactivation of β-catenin in EOC, and the involvement of this pathway in various aspects of EOC development.

### Overview of the Wnt/β-catenin signalling pathway

#### Wnt-off: inactivation and degradation of β-catenin

**Fig. 1:**
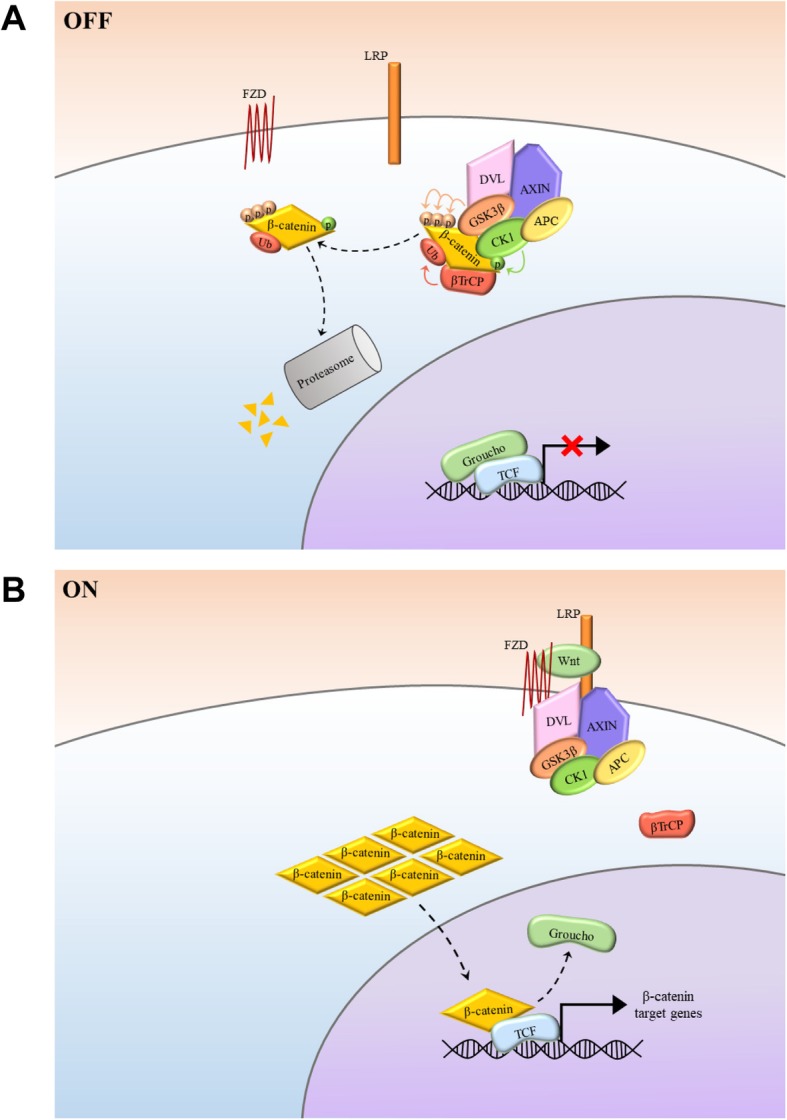
The Wnt/β-catenin signalling pathway. **a** Wnt signalling OFF. The absence of Wnt ligand binding to the FZD receptor prevents the interaction between FZD and LRP5/6. The destruction complex which resides in the cytoplasm binds to and promotes degradation of free cytoplasmic β-catenin. Specifically, CK1 and GSK-3β phosphorylate β-catenin, targeting it for βTrCP-mediated ubiquitination and subsequent proteasome degradation. Within the nucleus, the transcriptional repressor, Groucho binds to TCF and inhibits its transcriptional activity. **b** Wnt signalling ON. Binding of a Wnt ligand to FZD and LRP5/6 promotes the recruitment of DVL and the destruction complex to the membrane. As a result, the destruction complex’s ability to phosphorylate and degrade cytoplasmic β-catenin is inhibited. Cytoplasmic β-catenin accumulates and is translocated into the nucleus where it displaces Groucho and binds to TCF. Together with co-activators, the transcription of downstream target genes is initiated

β-catenin is the key mediator of the canonical Wnt pathway [9]. In the absence of a Wnt ligand, β-catenin is degraded by a destruction complex. The core components of this complex include AXIN, adenomatous polyposis coli (APC), casein kinase 1 (CK1), and glycogen synthase kinase 3β (GSK3β), as well as the E3 ligase, βTrCP (Fig. 1a). Protein phosphatase 2A (PP2A) is also associated with the β-catenin destruction complex. AXIN is a scaffolding protein that has interaction sites for multiple proteins including PP2A, APC, GSK3β, and CK1 [22]. Therefore, the presence of AXIN is essential for the assembly of the destruction complex. β-catenin is first phosphorylated by CK1 at the S45 and then by GSK3β at the S33, S37, and T41 [23, 24]. GSK3β also phosphorylates AXIN, stabilizing it and enhancing its interaction with β-catenin [25, 26]. APC, another core member of the destruction complex, contains multiple regions for AXIN and β-catenin interaction, enhancing β-catenin phosphorylation [22]. Finally, phosphorylated β-catenin is transferred to βTrCP, which forms a complex with Skp1 and Cullin to facilitate the ubiquitylation and degradation of β-catenin [27].

#### Wnt-on: activation of β-catenin

β-catenin signalling is activated in the presence of Wnt ligands. On the cell surface, the binding of a Wnt ligand induces the heterogeneous dimerization of Frizzled (FZD) and LRP5/6 receptors, leading to their conformation change [9]. Dishevelled (DVL) is then recruited to the membrane through its interaction with the cytoplasmic domain of FZD [28]. Here, DVL binds AXIN and facilitates the recruitment of the destruction complex to the membrane. The association between the destruction complex and the membrane is further strengthened following phosphorylation of the cytoplasmic domain of LRP5/6 by kinases including CDK14, and GSK3β [29]. Consequently, the activities of the destruction complex in promoting β-catenin phosphorylation and degradation are inhibited. Unphosphorylated cytoplasmic β-catenin can then accumulate and translocate to the nucleus. Since β-catenin does not have DNA-binding domain, it activates transcription through the association with TCF/LEF members, histone modifiers such as CREB-binding proteins (CBP), and other transcription factors [29]. Once inside the nucleus, β-catenin displaces the transcriptional repressor, Groucho, which forms a complex with TCF/LEF members in the absence of Wnt stimulation [13]. The active β-catenin/TCF complex can then initiate the transcription of its target genes [13] (Fig. 1b).

### Genetic alteration of the Wnt/β-catenin pathway in ovarian cancer

#### β-Catenin

The most common genetic alteration in the Wnt/β-catenin pathway involved in EOC is in the β-catenin gene, *CTNNB1* [21]. Mutations in this gene often result in an increased nuclear accumulation of β-catenin and, subsequently, an increase in transcription of its target genes [30]. This is most commonly observed in the EC subtypes, as one study found that activating mutations in *CTNNB1* accounted for up to 54% of the EC cases [21]. In ECs that carried a missense mutation in *CTNNB1*, the mutation was always found within the amino-terminal domain [21]. Phosphorylation of this domain by GSK3β is required for degradation of β-catenin, and therefore, mutations within this domain would render β-catenin resistant to degradation. Indeed, mutations within the GSK3β phosphorylation domain were positively correlated with the nuclear localization of β-catenin and the level of β-catenin/TCF target genes [31].

#### Destruction complex

Mutations in several components of the destruction complex, such as AXIN, GSK3β and APC, have been reported in EOC. Since these proteins are important for the degradation of β-catenin, genetic alterations that render them less effective or non-functional are likely candidates for driving hyperactive β-catenin signalling and, as a result, oncogenesis.

Although much less common than mutations in *CTNNB1*, mutations in the genes encoding AXIN and APC proteins (*AXIN1/2* and *APC*, respectively) have also been reported in EOC [21, 32]. AXIN protein exists in two isoforms: AXIN1 and AXIN2. A nonsense mutation in *AXIN1* has been found in one case of EC tumor, while a frameshift mutation in *AXIN2* resulting in truncation has been found in another EC tumor [21]. Functional analyses indicated that the frameshift mutation altered AXIN2 function and promoted β-catenin/TCF-dependent transcription [21].

Genetic alterations in APC, while frequently detected in colon cancers, are rarely found in EOC [11, 33]. As well, the involvement of *APC* mutations in EOC has been controversial. For instance, it was once believed that the I1307K missense mutation in the *APC* gene conferred a modest increase in the risk of hereditary and sporadic breast/ovarian cancer development through its association with BRCA1/2 mutations. Later analysis, however, concluded that, although there exists a high prevalence of I1307K mutation amongst BRCA1/2 carriers, the I1307K allele confers no additional risk for cancer development [34]. Two missense mutations (K90N, S1400L) and one nonsense mutation (R1114) within the *APC* gene were identified in an MC tumor [35]. While the exact contributions made by these mutations were not examined in this study, the APC variants were suggested to be likely involved in MC development. More research is needed to determine the mechanism underlying *APC* mutations and the frequency at which these mutations occur in EOC.

### Dysregulation of Wnt/β-catenin signalling in ovarian cancer

**Fig. 2:**
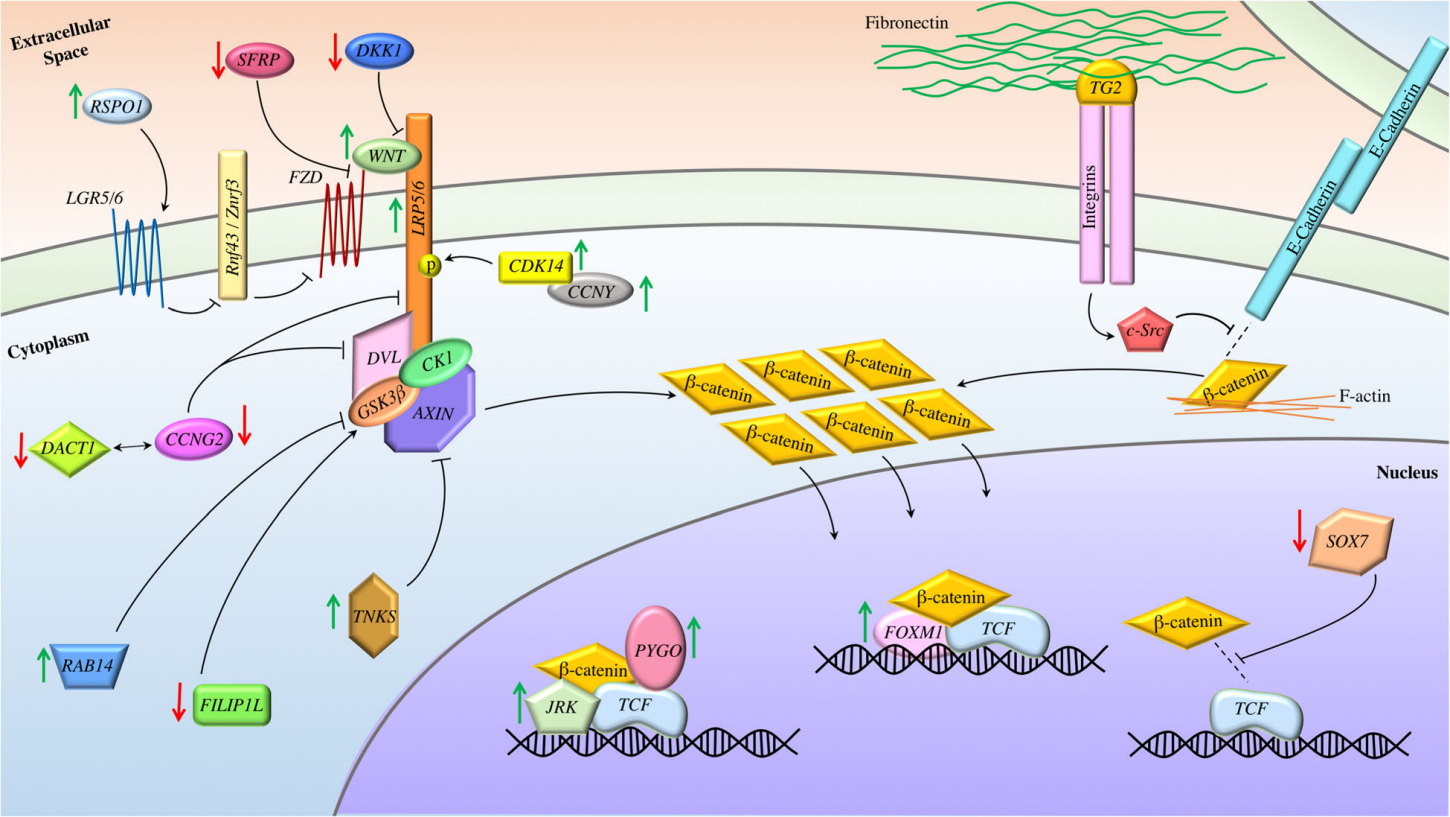
Proposed mechanisms of Wnt/β-catenin dysregulation in ovarian cancer. The Wnt/β-catenin pathway is regulated by many factors, whose aberrant expression leads to the hyperactivation of β-catenin in the EOC. Note that green arrows indicate proteins whose expression is upregulated in EOC, while red arrows indicate downregulation. DKK1 and SFRP2, which inhibit the dimerization of FZD and LRP5/6 and directly prevent FZD activation, respectively, are downregulated in EOC tumors. In contrast, Wnt ligands activate the pathway by forming a receptor complex with FZD and LRP5/6, while R-spondins bind LGRs and prevent the sequestration of the FZD. Both ligands and LGRs are overexpressed EOC. CCNY and CDK14 are also upregulated in EOC and have been suggested to work together to promote LRP5/6 phosphorylation and therefore activation. CCNG2, which is downregulated in EOC, decreases LPR6 and DVL levels. It may also interact with DACT1, also downregulated in EOC tumors, to promote DVL degradation. TNKS destabilizes AXIN to increase β-catenin activity and TNKS1 is known to be up-regulated in EOC. RAB14 inhibits the activity of GSK-3β and its upregulation contributes to higher β-catenin activity in EOC. FLIP1L, whose expression is negatively correlated with EOC progression, enhances GSK-3β activation in the destruction complex and is downregulated in EOC. This inhibition of the destruction complex results in the accumulation of β-catenin within the cytosol and its translocation into the nucleus. In addition, TG2, which is overexpressed in EOC, binds to integrin and fibronectin. This results in the recruitment of c-Src and disruption of E-cadherin/β-catenin complex on the membrane, which contributes to the accumulation of β-catenin within the cytoplasm. Finally, within the nucleus, higher expression of several co-activators of β-catenin/TCF, such as PYGO, JRK, and FOXM1, and lower expression of SOX7, which is known to inhibit the interaction between β-catenin and TCF, lead to the higher transcriptional activity of this complex

Although mutations in *CTNNB1* and components of the β-catenin destruction complex are rare or restricted to only the EC and MC subtypes, higher β-catenin activity is often observed in EOC, especially in HGSC. The mechanisms underlying the hyperactivation of the Wnt/β-catenin pathway in EOC are not entirely clear. However, many studies have reported the abnormal expression or activation of the components and regulators of this pathway. It is therefore highly possible that aberrant activities of these regulators contribute to the hyperactivation of Wnt/β-catenin in EOC, as summarized in Fig. 2 and discussed below.

#### Ligands and receptors

Several Wnt ligands have been reported to be upregulated and associated with poor prognosis in EOC patients. For example, using immunohistochemistry, Wnt-5A expression was found to be strongly upregulated in EOC tumors when compared with benign epithelial neoplasia and normal ovarian samples and was negatively correlated with patient survival [30]. This study also found higher Wnt-1 immunoreactivity in EOC tumors but no significant association between Wnt-1 expression and patient survival [30]. Another study, which measured mRNA levels of all Wnt ligands in ovarian tumors, revealed that Wnt-7A and Wnt-7B were highly expressed, while Wnt-3 and Wnt-4 were reduced, in malignant ovarian tissues compared with normal ovarian tissues [36]. Subsequent analyses of Wnt-7A by *in situ* hybridization confirmed that this up-regulation occurred more frequently in serous than in EC, MC, and CCC tumors [36]. Overexpression of Wnt-7A has also been identified in EC when compared to normal endometrium and benign endometrial lesions, and the immunoreactivity of Wnt7A in tumors was found to be negatively correlated with both overall and disease-free survival [37]. *In vitro* functional analysis showed that downregulation of Wnt-7A reduced ovarian cell proliferation, adhesion, invasion and expression of β-catenin target genes, suggesting an important role in the activation of Wnt/β-catenin signalling and EOC development [36]. The significance of Wnt-5A, Wnt-1 and Wnt-7B upregulation and Wnt-3 and Wnt-4 downregulation in EOC remains to be determined. Similarly, the causes of aberrant Wnt expression in EOC remain unclear and require further investigation.

A recent study revealed that abnormal expression of R-spondin 1 also contributes to the dysregulation of the Wnt/β-catenin signalling pathway at the receptor level [38]. R-spondin 1 (encoded by *RSPO1*) belongs to the secreted R-spondin protein family, which bind to the LGR4, LGR5 or LGR6 receptors [39]. This binding inhibits the sequestration of FZD by the transmembrane E3 ligases, Rnf43 and Znrf3 [39], thereby enhancing β-catenin activity [40]. A genome-wide association study identified SNPs at *RSPO1* as an EOC susceptibility locus [41]. RSPO1 was upregulated in all EOC cell lines and a small number of tissue samples when compared to normal ovarian tissue samples [38]. In HSGC subtype ovarian tumors, upregulation of *RSPO1*, *RSPO2*, *LGR5* and *LGR6* expression, although at low frequency, has also been observed [42]. Moreover, overexpression of RSPO1 increased EOC cell proliferation, migration, and chemoresistance [38]. Furthermore, overexpression of RSPO1 enhanced, whereas deletion of RSPO1 attenuated, β-catenin activity [38]. Similarly, silencing of LGR6 inhibited β-catenin activity [43]. These findings strongly support the role of RSPO1/LGR in enhancing Wnt/β-catenin signalling and suggest that their upregulation during EOC development contributes to the hyperactive β-catenin signalling.

Wnt/β-catenin signalling is tightly controlled by several negative regulators, some of which inhibit activation of Wnt signalling by competing with Wnt ligands for their receptors. Abnormal levels of these regulatory proteins have been associated with the dysregulation of Wnt/β-catenin activity and EOC development. For example, Dickkopf (DKK) inhibits Wnt signalling by binding to LRP5/6 and disrupting the dimerization of FZD and LRP5/6 [44]. Interestingly, DKK1 was reported to be downregulated in EOC cells and negatively correlated with the stage of tumor development [45]. The expression of DKK2 was also significantly lower in EOC tumors than in normal ovary [46]. Secreted Frizzled-related proteins (SFRPs), similarly, interact with Wnt ligands and FZD receptors to abrogate their activations [44]. SFRP4 expression has been observed to be significantly downregulated in EOC cell lines and to be negatively correlated with the aggressiveness of EOC tumors and patient survival [47, 48]. In OVCAR3, a cell line with no detectable levels of SFRP4 [47], treatment with SFRP4 strongly inhibited β-catenin activity [49], indicating the important role of SFRP4 in suppressing β-catenin activity.

Downregulation of DKK2 in EOC tumors is believed to involve epigenetic silencing. Compared to benign tumors and normal ovarian tissues, DKK2 in EOC tumors was more commonly methylated and its methylation was increased in higher grades and stages of EOC [46]. Similarly, decreases in DKK1 and SFRP2 levels in EOC have been suggested to be induced by DNA methylation [50]. TET1, a member of ten-eleven translocation (TET) family, was reported to promote the activation of DKK1 and SFRP2 expression in EOC [50]. TET1 plays an essential role in DNA demethylation by catalytically converting 5-methylcytosine to hydroxymethylcytosine, 5-formylcytosine and 5-carboxylcytosine [51]. Hypomethylation of the DKK1 and SFRP2 promoters was observed in EOC cells with ectopic TET1 expression [50]. Therefore, TET1 may bind to the CpG islands at the promoter regions of DKK1 and SFRP2, reducing the methylation levels and stimulating their expression by the transcriptional machinery. This is supported by the increase in DKK1 and SFRP2 levels observed in EOC cells with induced expression of TET1 and by undetectable levels of TET1 expression in several EOC cell lines [50]. Furthermore, the downregulation of TET1 is correlated with the clinical stage in EOC tissues [50].

Cyclin G2 (CCNG2) is an unconventional cyclin which has been shown to inhibit cell proliferation, migration and invasion in EOC cells [52, 53]. CCNG2 was downregulated in EOC tissues compared to tumors with low malignant potential or normal ovarian tissues [53]. The inhibitory effects of cyclin G2 on EOC cell proliferation and invasion are mediated, at least in part, by the inhibition of β-catenin. Specifically, CCNG2 has been found to reduce LRP6, DVL2, and β-catenin levels in EOC [53]. While the mechanism by which CCNG2 inhibits LPR6 and DVL2 in EOC is not known, a recent report in gastric cancer indicated that CCNG2 downregulated DVL2 through the interaction with Dapper1 (DACT1) [54], a Wnt signalling antagonist that has been shown to promote DVL2 degradation [55]. Gao *et al.* revealed that there was a direct interaction between CCNG2 and DACT1 and that overexpression of DACT1 decreased DVL2 and β-catenin levels in gastric cancer cells [54]. While unphosphorylated DACT1 inhibited Wnt/β-catenin pathway, its phosphorylated form promoted Wnt/β-catenin signalling [56]. Remarkably, overexpression of CCNG2 inhibited phosphorylation of DACT1 by CK1, suggesting CCNG2 exhibits the inhibitory effects on canonical Wnt signalling by suppressing DACT1 phosphorylation through direct interaction and promoting DVL2 degradation by unphosphorylated DACT1 [54]. Recently, it was reported that DACT1 was downregulated in EOC samples derived from LGSC, EC, CC, and MC, when compared with ovarian tissues collected from patients with benign gynecological disorders [57]. Thus, down-regulation of CCNG2 and DACT1 could contribute to the hyperactivation of the Wnt/β-catenin pathway; however, whether or not there is an interaction between CCNG2 and DACT1 in EOC requires further investigation.

Another cell cycle regulator, cyclin Y (CCNY), also regulates β-catenin signalling. CCNY has been found to be upregulated in EOC tissues and its expression to be positively correlated with the clinicopathological stage [58]. In addition, the overexpression of CCNY increased cell proliferation, migration, and invasion, which was mediated by the Wnt/β-catenin pathway. Ectopic expression of CCNY increased nuclear β-catenin levels and its transcriptional activity, leading to the upregulation of downstream target genes. A previous study indicated that cyclin Y and CDK14 could interact at the membrane to modulate LRP6 activation through phosphorylation [59]. Notably, the expression of CDK14 was also upregulated in clinical EOC samples and its expression was found to enhance the accumulation of nuclear β-catenin [60]. Therefore, the upregulation and association of cyclin Y and CDK14 in EOC may promote canonical Wnt signalling.

#### The β-catenin destruction complex

Decreases in the expression of certain components of the destruction complex are frequently observed in EOC [61]. For example, several studies have reported significantly higher methylation rates in the promoter region of *APC* in EOC tumors when compared to benign ovarian tumors or normal ovarian tissue samples [62–64]. However, the mechanisms underlying the hypermethylation and suppression of APC in EOC are not clear.

Tankyrases (TNKS), which belong to the poly (ADP-ribose) polymerase (PARP) family, are positive regulators of Wnt/β-catenin signalling [65, 66]. TNKS catalyzes ADP-ribosylation of AXIN and destabilize the protein. Upregulation of TNKS1 expression was observed in EOC tissues and the immunoreactivity of TNKS1 was positively correlated with tumor size and stage [67]. Furthermore, inhibition or knockdown of TNKS1 reduced EOC cell proliferation, migration, invasion, and colony formation *in vitro* and tumor growth in nude mice, as well as aerobic glycolysis. Further studies confirmed that TNKS1 exerts these effects by promoting Wnt/β-catenin signalling [67].

Inhibition of GSK3β has also been observed in EOC [68, 69]. Initially, it was reported that GSK3β was overexpressed in EOC and was positively regulated the proliferation of ovarian cancer cells [61, 70]. However, further analyses revealed that GSK3β was frequently phosphorylated and thereby inactivated in EOC [12]. It was then postulated that GSK3β phosphorylation may be linked to the high frequency of activating mutations in PI3K in ovarian cancers [12]. The PI3K/AKT pathway is known to inhibit GSK3β activity through phosphorylation of S9 [71]. This increase in PI3K results in higher levels of active AKT, which in turn inactivates GSK3β and thus downregulates β-catenin signalling. In addition, Filamin A interacting protein 1-like (FILIP1L), which was reported to be down-regulated in EOC and negatively correlated with EOC tumor stages, chemoresistance, and patient survival [69], has been found to induce β-catenin degradation [69, 72]. While the underlying mechanism by which FILIP1L inhibits β-catenin has not been determined in EOC, knockdown of FILIP1L in colon cancer cell lines led to an increase in phosphorylated AKT and GSK-3β and a decrease in phosphorylated β-catenin levels, suggesting that FILIP1L may promote β-catenin degradation by inhibiting AKT and thereby increasing GSK3β activity [73]. Finally, RAB14, a member of the RAS small G-protein superfamily [68, 74], has also been reported to be upregulated in EOC tissues and cell lines [68]. Overexpression of RAB14 increased GSK3β phosphorylation at S9 and enhanced β-catenin activity [68], suggesting that higher expression of RAB14 in EOC tumors contributes the hyperactivation of β-catenin by inhibiting GSK3β activity.

#### Regulation of β-catenin subcellular localization

β-catenin is a dynamic protein that can function as a component of adherens junctions or as a transcription factor depending on its subcellular localization. At the adherens junctions, β-catenin interacts with the cytoplasmic tail of E-cadherin and links E-cadherin to actin filaments through its interaction with α-catenin to maintain the dynamics of the cytoskeleton [75, 76]. Dissociation of the adherens junctions results in the accumulation of β-catenin in the cytoplasm and its nuclear translocation to promote transcription of target genes [77]. The dissociation between β-catenin and E-cadherin is mediated by tyrosine phosphorylation at the C-terminal of β-catenin, decreasing its binding affinity to E-cadherin and α-catenin [78]. In contrast, serine phosphorylation of E-catenin at its cytoplasmic tail increases the binding between E-cadherin and β-catenin [75], stabilizing the adherens junctions complex.

During EOC development, the membrane-associated β-catenin is dysregulated. Tissue transglutaminase 2 (TG2) has been shown to promote the dissociation of E-cadherin and β-catenin in EOC cells. TG2 was found to be overexpressed in EOC tumors and positively correlated with β-catenin levels in ovarian cancer cell lines [79, 80]. TG2 forms a complex with fibronectin (FN) and β1-integrin, enhancing the binding of FN to its cognate receptor and leading to the activation of c-Src. It has been proposed that, at the plasma membrane, activated c-Src phosphorylates β-catenin on Tyr 654, thereby inhibiting its interaction between E-cadherin [80]. Finally, activation of lysophosphatidic acid receptors (LPAR) by its ligand, lysophosphatitic acid (LPA) which is abundantly present in the ascites of EOC patients, also contributes to the loss of membrane β-catenin, probably by activating β1-integrin and promoting the recruitment of E-catenin to the β1-integrin clusters [81].

#### Regulation of β-catenin in the nucleus

Several proteins are believed to modulate β-catenin activity within the nucleus. SOX7, a member of the Sox transcription factor family, was demonstrated to suppress Wnt signalling in ovarian cancer cells harboring either wildtype or mutant β-catenin [82]. This was proposed to be accomplished through direct binding of SOX7 to β-catenin to disrupt its activity. Ectopic expression of SOX7 in TOV-112D cells significantly inhibited β-catenin transcriptional activity with downregulation of β-catenin/TCF target genes. Immunofluorescence and co-immunoprecipitation analysis indicated that SOX7 mainly localized in the nucleus where it interacted with β-catenin and TCF4 [82]. Furthermore, SOX7 expression was found to be significantly reduced in EOC tumors and negatively correlated with tumor progression [83]. Bioinformatics analyses predicted that another member of the Sox family, SOX17, would be involved in the development of ovarian cancer through its interaction with β-catenin [84]. However, this has not yet been proven experimentally.

Multiple proteins have been reported to form a complex with β-catenin and TCF/LEF and increase the stability of the transcriptional complex. Pygopus (PYGO) binds β-catenin directly in the nucleus and assists in transcription of its target genes [32]. PYGO2 has been detected in the all histological subtypes of EOC tumors and its expression was higher in EOC tissues than in benign ovarian tumors [85]. Suppression of PYGO2 inhibited cell proliferation, colony formation, and tumor growth, suggesting that it promotes ovarian cancer progression [85]. However, whether or not PYGO2 exerts these tumor-promoting effects by promoting β-catenin/TCF activity requires further confirmation. In addition, JRK, which interacts directly with β-catenin through its N-terminal, stabilizes the transcriptional complex consisting of β-catenin, LEF1, and PYGO2 [86, 87]. Mining the TCGA database revealed copy-number gains in JRK and higher JRK mRNA levels in some serous tumors [87]. Furthermore, knockdown or deletion of the N-terminal of JRK decreased the activity of β-catenin, downregulated β-catenin target genes, and inhibited cell proliferation [87]. These findings suggest that JRK promotes EOC development by enhancing β-catenin activity.

FOXM1, a member of forkhead transcription factors, plays important roles in EOC development in part by regulating β-catenin signalling. High FOXM1 levels were found to be correlated with EOC tumor grade and stage, and to predict poor prognosis and chemoresistance [88–91]. Interestingly, high FOXM1 immunoreactivity was significantly associated with high β-catenin staining [92]. FOXM1 has been shown to induce β-catenin transcription in EOC cells [91, 92]. In addition, FOXM1 has also been reported to promote β-catenin nuclear translocation and to form a complex with β-catenin and TCF4 to induce target gene expression in glioma cells [93]. On the other hand, activation of Wnt/β-catenin signalling also increased mRNA and protein levels of FOXM1 [91]. The findings suggest that FOXM1 and β-catenin form a positive feedback loop that contributes to EOC development.

#### Role of non-coding RNAs in regulating the β-catenin activity

Non-coding RNAs are RNA transcripts that do not encode proteins [94–96]. However, they are important regulatory molecules that modulate cellular processes by controlling gene expression. There are three major types of regulatory non-coding RNAs: long non-coding RNA (lncRNAs), microRNAs (miRNAs) and circular RNAs (circRNAs). lncRNAs are transcripts which have the length greater than 200 nucleotides while miRNAs are transcripts with 30 nucleotides or shorter [94, 95]. lncRNAs exhibit a broad range of mechanisms in mediating transcriptional repression or activation due to interactions with both RNAs and proteins [94]. In contrast, miRNAs suppress gene expression primarily through complementary binding to their target mRNAs, inhibiting their translational activity and reducing the stability of the target transcripts [96]. circRNAs are generated through the process of back splicing and function as miRNA sponges and protein scaffolds to regulate gene expression [97]. Aberrant expression of non-coding RNAs is implicated in ovarian tumorigenesis.

Multiple studies have shown the involvement of non-coding RNAs in EOC progression through the modulation of the Wnt/β-catenin pathway (summarized in Tables 1 and 2). Dysregulation of miRNAs promotes EOC pathology in many ways including proliferation, metastasis, and chemoresistance [95]. For example, miR-92a, miR-939, and miR-1207 were upregulated in EOC and promoted Wnt/β-catenin signalling via direct repression of Wnt inhibitors including DKK1, APC2, SFRP1, AXIN2 and ICAT [100, 103, 104]. Similarly, miR-126-5p targeted negative regulators of the Wnt/β-catenin pathway, namely DKK3 and AXIN1 [99]. In contrast, miRNAs such as miR-15b and miR-219-5p, which regulate the expression of various Wnt components including Wnt7A and Twist, have been reported to be downregulated in EOC, allowing the upregulation of Wnt signalling at various levels [111, 116].

**Table 1:** Regulation of the Wnt/β-catenin signaling pathway by miRNAs in EOC

miRNA	Targets	Expression in EOC	Effects on EOC	Effects on β-catenin activity	Reference
miR-27a	*FOXO1*	Increased	Promote EMT in ovarian cancer	Activate	[98]
miR-126-5p	*AXIN1, DKK3*	N.D	Promote platinum resistance	Activate	[99]
miR-92a	*DKK1*	Increased	Promote stemness and chemoresistance	Activate	[100]
miR-762	*MEN1*	Increased	Promote proliferation, migration and invasion and inhibit apoptosis	Activate	[101]
miR-197	*NLK*	Increased	Promote taxol resistance	Activate	[102]
miR-939	*APC2*	Increased	Promote proliferation and anchorage-independent growth	Activate	[103]
miR-1207	*SFRP1, AXIN2, ICAT*	Increased	Promote cancer stem-like trait	Activate	[104]
miR-16	ND	Decreased	Inhibit proliferation, migration and invasion	Suppress	[105]
miR-340	*FHL2*	Decreased	Inhibit proliferation and metastasis	Suppress	[106]
miR-34c	*SOX9*	Decreased	Inhibit proliferation and cisplatin chemoresistance	Suppress	[107]
miR-377	*CUL4A*	Decreased	Inhibit cell proliferation	Suppress	[108]
miR-370	*FOXM1*	Decreased	Inhibit proliferation and metastasis	Suppress	[109]
miR-214	ND	Decreased	Inhibit proliferation and invasion	Suppress	[110]
miR-219-5p	*TWIST1*	Decreased	Inhibit proliferation, migration, and invasion	Suppress	[111]
miR-152	*WNT1, ADAM17*	Decreased	Inhibit EMT, migration, and invasion	Suppress	[112]
miR-133a-3p	ND	Decreased	Inhibit proliferation and invasion	Suppress	[113]
miR-429	*KIAA0101*	Decreased	Inhibit cell migration, invasion and cisplatin resistance	Suppress	[114]
miR-381	*YY1*	Decreased	Inhibit proliferation and migration	Suppress	[115]
miR-15b	*WNT7A*	Decreased	Decrease adhesion and invasion	Suppress	[116]
miR-101	*MARCH7, ZEB1, ZEB2*	Decreased	Inhibit proliferation, migration, and invasion	Suppress	[117–119]

**Table 2:** Wnt/β-catenin pathway-associated long noncoding RNAs in EOC

	Targets	Expression in EOC	Effects on EOC	Effects on β-catenin activity	Reference
Long noncoding RNAs					
CCAT2	ND^*^	Increased	Promote EMT	Activate	[120]
SNHG20	Inactivate GSK3b	Increased	Promote proliferation and inhibit apoptosis	Activate	[121]
MALAT1	Increase DVL2 and β-catenin	Increased	Promote proliferation, migration and inhibit apoptosis	Activate	[122]
Linc-ROR	ND	Increased	Promote proliferation, migration and invasion through EMT	Activate	[123]
HOTAIR	ND	Increased	Promote proliferation and chemoresistance	Activate	[124]
HOXD-AS1	miR-133a-3p, miR-186-5p	Increased	Promote cell proliferation and invasion	Activate	[113]
circRNAs					
Circ-ITCH	miR-145	Decreased	Inhibit OC cells proliferation, migration and invasion	Suppress	[125]
Circ_0061140	miR-370	Increased	Promote cell proliferation and migration	Activate	[109]

^*^ND, not determined

In addition to modulating the expression of key components of the Wnt/β-catenin pathway, many miRNAs have been reported to inhibit regulators of this pathway. Recently, we demonstrated that miR-590-3p levels were upregulated in EOC tissues when compared to normal ovarian tissue and EOC tumors with low malignancy potential [126]. miR-590-5p enhanced cell proliferation, invasion and migration *in vitro,* and promoted tumor formation and metastasis *in vivo* [127]. We showed that miR-590-3p targeted CCNG2 and FOXO3, a transcription factor that induces CCNG2 transcription in EOC, and enhanced β-catenin activity [127, 128]. Silencing of *CTNNB1* attenuated the effect of miR-590-3p-induced formation of compact spheroids, indicating that miR-590-3p promotes EOC development in part via the activation of Wnt/β-catenin signalling [127]. Several other miRNAs that modulate the activity of β-catenin, are also dysregulated in EOC. It was reported that miR-340 was down-regulated in EOC and exerted anti-tumor effects by targeting four and a half LIM domain protein 2 (FHL2), a co-activator of β-catenin [106]. Another study reported that miR-762 promoted EOC cell proliferation, migration, and invasion by upregulating Wnt/β-catenin signalling via suppression of menin [101], which has been reported to promote β-catenin cytoplasmic shuttling and degradation [129, 130]. Finally, miR-377, miR-101, miR-381, and miR-429 were found to target Cullin E3-Ring E3-ligase family member, CUL4A, membrane-associated E3 ubiquitin ligase MARCH7, transcription factor Ying Yang 1 (YY1), and the PNCA-associated factor, KIAA0101, respectively, in EOC [108, 114, 115, 117]. Ectopic expression of CUL4A, MARCH7, YY1, and KIAA0101 promoted β-catenin nuclear translocation and downstream target gene expression. However, whether there is a direct interaction between CUL4A, MARCH7, YY1, KIAA0101 and central Wnt regulators in EOC remains to be elucidated.

Several lncRNAs have been reported to be upregulated in EOC, activate β-catenin signalling, and promote EOC development (Table 2). However, little is known about how these lncRNAs promote β-catenin signalling. For example, HOXD-AS1 has been found to upregulate β-catenin by down-regulating two miRNAs that target the Wnt/β-catenin pathway. HOXD-AS1 was overexpressed in EOC tumors and negatively correlated with patient survival [131]. HOXD-AS1 was found to directly bind to miR-186-5p, which targeted PIK3R3, a regulatory subunit of PI3K [131]. Although the consequence of miR-186-5p inactivation by HOXD-AS1 on β-catenin has not been determined in EOC cells, it was reported that miR-186-5p enhanced AKT phosphorylation and β-catenin levels in prostate cancer cells [132]. HOXD-AS1 was also reported to target miR-133a-3p and to increase Wnt/β-catenin signalling [113]. However, it is unclear if and how these two events are related. It has been reported that SNHG20 induced GSK3β inactivation [121], whereas MALAT1 increased DVL2 and β-catenin levels [122]; however, the underlying mechanisms of their actions are still elusive.

While modulation of β-catenin signalling by circRNAs has been reported in other types of cancer [133–136], there are currently no direct evidence that circRNAs affect EOC development through regulation of the Wnt/β-catenin pathway. However, a recent study has shown that circ_0061140 exerts tumor-promoting effects by sponging miR-370, which targets FOXM1 [109]. Since FOXM1 enhances β-catenin signalling [91, 93], it is possible that circ_0061140 would also increase the activity of β-catenin. In addition, downregulation of circ-ITCH has been observed in EOC tissues [125]. It has been reported in colorectal and lung cancer that overexpression of circ-ITCH inhibited β-catenin expression and its transcriptional activity, suppressing cancer cell proliferation [137, 138]. Mechanistically, circ-ITCH sponged miR-22-3p to regulate CBL levels in thyroid cancer cells [139]. CBL is a unique E3 ligase that can translocate into the nucleus with β-catenin and modulate nuclear β-catenin degradation in the Wnt-on phase [139]. Therefore, there is an association between circ-ITCH and Wnt/β-catenin in carcinogenesis, but whether or not this occurs in EOC remains to be investigated.

### Role of Wnt/β-catenin in ovarian cancer development

It is well established that the Wnt/β-catenin pathway exerts tumor-promoting effects in EOC [12, 94, 95]. This pathway has been shown to promote cell proliferation, survival migration, and invasion, maintain cancer stem cells, induce resistance to therapeutic agents, and may also be involved in the tumor angiogenesis [18] and immune suppression [19].

#### Stemness

It is now widely accepted that tumors are made up of a heterogeneous population of cancer cells, a small portion of which is characterized as cancer stem cells (CSCs) [140]. Like normal stem cells, CSCs possess self-renewal and differentiation potential that contribute to the heterogeneity of cancer cell populations. CSCs have high tumorigenic potential and play major roles in driving tumor initiation, metastasis, chemoresistance, and cancer recurrence [140]. Ovarian CSCs have been characterized by functional and phenotypic expression of surface markers such as CD24, CD44, CD117, ALDH, CD133, SOX2, NANOG, OCT4 and EPCAM [91, 104, 141, 142]. Ovarian tumor-isolated mesenchymal stem cells were identified to exhibit high levels of CD133 and ALDH expression [143]. Additionally, the increase in stem cell marker expression in ovarian CSCs was detected together with the ability to form spheroids *in vitro* and tumors *in vivo*, contributing to the initiation and progression of EOC [91, 100, 104, 143]. These cells are more resistant to chemotherapy and capable of giving rise to progenitor tumor cells, leading to tumor progression, metastasis, and recurrence [144–149].

Accumulating evidence points toward the Wnt/β-catenin pathway in playing an important role in the acquisition of stem-like properties in ovarian cancer cells [91, 104, 150]. Among stem cell markers, ALDH1A1 has been found to be a direct transcriptional target of β-catenin [151]. In addition, silencing of β-catenin strongly reduced the stem-like properties [17, 151]. These findings provide direct evidence that β-catenin is involved in promoting EOC stemness.

Several studies have found that modulation of β-catenin activity altered the CSC-like properties. For example, miR-1207 suppressed SFRP1, AXIN2, and ICAT, three important negative regulators of the Wnt/β-catenin signalling pathway, to activate β-catenin signalling and promote the expression of CSC markers [104]. Likewise, Wnt positive regulators LGR5 and LGR6 have been recognized as markers for ovarian cancer stem cells [43, 152, 153]. The high expression of LGR5 and LGR6 was positively correlated with poor patient survival and was observed predominantly in high-grade tumors [43, 152]. Silencing of LGR6 significantly inhibited stemness and the effects of LGR6 were demonstrated to be mediated by the β-catenin activity [43]. Finally, several β-catenin inhibitors were documented to exert inhibitory effects on ovarian CSCs. Theaflavin-3, 3'-digallate (TF3), a black tea polyphenol, was found to inhibit EOC stemness by blocking Wnt/β-catenin signalling [154]. Ginsenoside-Rb1, a natural saponin isolated from the rhizome of *Panax quinquefolius* and *notoginseng*, and its metabolite, compound K, suppressed CSC self-renewal and inhibited β-catenin activity [155]. Together, these studies strongly support the critical role of the Wnt/β-catenin pathway in maintaining stemness in EOC.

#### Chemoresistance

Recent studies encompassing ovarian CSCs and their involvement in EOC tumorigenesis reveal the association of CSCs and chemoresistance [91, 100, 156, 157]. There is a high correlation between nuclear β-catenin levels/activities and chemoresistance of stem-like EOC cells. ALDH^+^/CD44^+^ ovarian CSCs exhibited higher levels of resistance to paclitaxel and carboplatin [157]. Additionally, an increase in cisplatin and paclitaxel resistance was observed in IGROV1 sublines and was associated with elongated mesenchymal-like morphology and a decrease in cell-cell interactions [91]. Since CSCs are linked to chemoresistance, many of the studies described above also reported the chemosensitizing effects of β-catenin silencing [158] or inhibition [38, 67, 91] on EOC cells.

One of the mechanisms for chemoresistance in CSCs is the deregulation of membrane transporters, such as an ATP-binding cassette (ABC) transporter, ABCG2 [91, 104, 159]. The study by Chau et al. (2013) identified the involvement of c-kit (also known as CD177), a stem cell-associated receptor tyrosine kinase, in promoting ovarian stem-like phenotypes and chemoresistance via the Wnt/β-catenin/ABCG2 axis [159]. c-kit and SCF were upregulated in ovarian tumor-initiating cells. Knockdown of c-kit reduced the numbers of spheroids formed *in vitro* and rendered the cells more susceptible to chemotherapeutic reagents, including cisplatin and paclitaxel [159]. In addition, increased c-kit transcriptional level led to an increase in Wnt/β-catenin signalling pathway and mRNA levels of ABCG2 transporter, which promoted the efflux of chemotherapeutic drugs as the results [159]. Besides, Wnt/β-catenin pathway was reported to indirectly modulate the expression of human copper transporter 1 (hCRT1) via FOXM1 [91]. hCRT1 is a transmembrane transporter that allows the passage of copper and cisplatin through the membrane barrier into cells [91]. In cisplatin-resistant EOC cells, upregulation of FOXM1 inhibited the expression of human copper transporter 1 (hCTR1) and SP1, a transcription factor that induces hCTR1 expression [91]. It has been demonstrated that FOXM1 promotes β-catenin nuclear localization while β-catenin activation promotes FOXM1 expression as a positive feedback loop [91, 93]. In response to Wnt-3A, FOXM1 expression was upregulated in TOV-21G cells in a dose-dependent manner [91]. Thus, induced expression of FOXM1 by Wnt/β-catenin signalling would impair cisplatin uptake in EOC cells.

In addition to promoting resistance to conventional chemotherapies, a recent study provided evidence to support the activation of the Wnt/β-catenin signalling in inducing resistance to a PARP inhibitor, olaparib [160]. Activators and target genes of the Wnt/β-catenin pathway were found to be induced, while inhibitors of this pathway were suppressed in olaparib-resistant HGSC cell lines. Overexpression of Wnt-3A reduced the sensitivity of EOC cells to olaparib. Conversely, inhibition of Wnt/β-catenin signalling enhanced the anti-tumor effects of olaparib both *in vitro* and *in vivo* [160]*.* These results and studies discussed above support the potential of Wnt/β-catenin inhibitors for the management of EOC patients with drug resistance.

#### EMT and metastasis

Epithelial to mesenchymal transition (EMT) is a cellular process in which epithelial cells lose cell-cell adhesion and acquire mesenchymal characteristics, including migration and invasion [161]. The attainment of invasiveness allows cells to break through the basement membrane, which eventually results in metastasis in ovarian cancer [12, 161]. Many studies examining invasive characteristics of ovarian cancer suggest that activation of EMT is a critical step in acquiring malignant phenotypes, especially in high-grade serous ovarian carcinoma [162, 163].

Recent evidence indicates that the activity and expression levels of E-cadherin and β-catenin are critical in the initiation of EMT in ovarian cancer cells [162]. Loss of E-cadherin has been observed in ovarian cancer cell lines with increased invasion and migration phenotypes [123, 164]. E-cadherin assists in keeping a low cytosolic/nuclear β-catenin level by forming a complex with β-catenin at the adherens junctions and, therefore, the decrease in E-cadherin would involve in the promotion of β-catenin signalling. The accumulation of nuclear β-catenin levels was detected together with decreased levels of E-cadherin and increased cancer cell motility [113, 164]. Moreover, the Wnt/β-catenin pathway modulates the expression of E-cadherin through upregulation of key transcription factors, whether directly or indirectly. These transcription factors are known as mesenchymal inducers, and include Twist, Snail and Slug [98, 105, 113, 130, 163, 165, 166]. Twist, Snail and Slug bind to specific E-boxes located proximal to the E-cadherin promoter and suppress its expression [167]. In addition, Snail can form a transcriptional complex with β-catenin, providing a positive regulatory feedback to enhance its own expression through the transcriptional activity of β-catenin [167, 168].

Additionally, Wnt/β-catenin signalling is involved in the remodeling of the extracellular tumor matrix in EOC, which is suggested to be mediated by the activities of matrix metallopeptidases (MMP). MMPs are proteolytic enzymes that act on diverse extracellular matrix (ECM) components such as fibronectin, gelatins, collagens, and laminins [169]. MMP-2, MMP-7, and MMP-9 have all been shown to be upregulated in Wnt-activated cells and were reported as direct transcriptional targets of β-catenin [36, 101, 123, 170]. Dysregulation of these MMPs was frequently observed in EOC [171–173]. MMP-9 [174] and MMP-2 [164] promoted invasion and metastasis while MMP-7 was reported to activate MMP-2 and MMP-9 *in vitro* [175]. Furthermore, increased β-catenin levels have been detected in tumor samples from orthotopic xenograft mice implanted with high metastatic EOC cells [176]. Silencing of β-catenin displayed a significant reduction in the ability to form primary tumors and ascites in the mouse model, providing direct evidence for an essential role of β-catenin in EOC metastasis [176].

#### Tumor angiogenesis

Tumor angiogenesis, wherein tumors promote blood vessel formation to provide themselves with nutrients and oxygen, is one of the hallmarks of cancer [16]. Multiple steps are involved in angiogenesis including vasculature disruption, cell migration, cell proliferation, and vessel formation [177]. While studies in other cancers have provided strong evidence that the Wnt/β-catenin pathway is an important player in tumor angiogenesis [16, 178], very few studies have been done in EOC. A recent study by Tang *et al.* (2018) examined the role of soluble E-cadherin in EOC and revealed that it interacted with VE-cadherin to induce angiogenesis [18]. Interestingly, soluble E-cadherin containing exosomes induced strong β-catenin accumulation in the nucleus. Importantly, silencing of β-catenin expression attenuated the effect of soluble E-cadherin containing exosomes on the formation of network-like structure [18]. These findings suggest that β-catenin may induce tumor angiogenesis. However, more studies, especially involving *in vivo* mouse models, is required to confirm the role of β-catenin in ovarian tumor angiogenesis.

#### Immune suppression

Ovarian cancer has been reported to evade the immune system using multiple mechanisms, including the recruitment of regulatory T cells (Treg) and the promotion of T cell apoptosis via PD-L1 [179, 180]. The presence of Treg in ovarian tumors increases immune tolerance and is correlated with poor patient prognosis [179]. In addition, IL-10 and indoleamine 2, 3-dioxygenase (IDO) were reported to promote immune evasion by ovarian tumor-associated macrophages [181]. Notably, expression of IDO is associated with poor prognosis in ovarian cancer [182, 183]. In the presence of ovarian tumor ascites CD14^+^ cells, which expressed IDO and IL-10, CD4^+^ T cells showed inhibition in responsiveness to antigen stimulation, suggesting IDO and IL-10 might be involved in the regulation of the immune response in EOC [181]. The same study suggests that IDO may induce Treg differentiation and apoptosis of T-cells, regulating the balance of Treg and effector T cells Th17. IDO promoter contains TCF/LEF binding domains, which was reported to be activated by Wnt/β-catenin signaling [184]. However, there is no direct evidence indicating the Wnt/β-catenin pathway promotes immune evasion of EOC cells.

## Conclusion and future perspectives

Aberrant Wnt/β-catenin signalling has been widely linked to cancer development. Increasing evidence indicates that this pathway is hyperactivated in EOC and plays important roles in driving EOC development. Although mutations of *CTNNB1*, *APC*, and *AXINs* are restricted to the EC and MC subtypes of the EOC, hyperactivation of β-catenin is commonly observed in HGSC tumors and involves diverse mechanisms. This could be due to overexpression of ligands and receptors, underexpression of inhibitors of the Wnt/β-catenin pathway, and altered expression of proteins that regulate β-catenin/E-cadherin interaction on the membrane or β-catenin/TCF transcriptional activity, as summarized in Fig. 2. In addition, many non-coding RNAs, particularly miRNAs, have been shown to modulate this pathway, directly and indirectly, to exert their oncogenic or anti-tumor effects on EOC. The role of circRNAs in EOC development is emerging; however, little is known about how circRNAs modulate β-catenin signalling in EOC.

Compared to other types of cancer, especially colon cancer, fewer studies have been done on EOC to investigate the roles and mechanisms of the Wnt/β-catenin signalling pathway in the process of tumorigenesis. Nevertheless, evidence accumulated to date strongly supports a critical role for this pathway in promoting several key aspects of EOC development, from promoting CSC self-renewal, EMT and metastasis, drug resistance, and tumor angiogenesis, to suppressing tumor immunity. However, many of these studies were carried out using established cell lines and some of them were only conducted *in vitro*. Therefore, it is important to confirm key findings in primary tumor cells collected from patients. Furthermore, EOC is composed of different histologic subtypes, each with distinct molecular features, mutational profiles, and even cellular origins [3]. While the tumor-promoting effects of β-catenin have been observed in cell lines representing different subtypes of EOC, further comprehensive comparisons regarding the actions of β-catenin among different subtypes would provide an insight into the contribution of this signaling pathway in the pathogenesis of each subtype. Also, more studies are required to further investigate the functions and mechanisms of the Wnt/β-catenin pathway in promoting tumor angiogenesis and immune evasion.

The Wnt/β-catenin pathway is recognized as an important target for cancer therapy and many studies have been done to investigate the potential therapeutic effects of antibodies and small molecules that target this pathway and some of them are currently in clinical trials [185–188]. However, most of these studies focus on other types of cancer, especially colon cancer. Several studies carried out on EOC cells have shown that inhibitors of this pathway strongly reduced tumor growth and metastasis [100, 189, 190]. To date, only one clinical study on Wnt targeting drugs has been reported for EOC. In a phase 1b clinical trial, ipafricept, a fusion protein that antagonizes Wnt signalling by binding Wnt ligands, was found to be well tolerated when used with standard chemotherapies [191]. Given the hyperactivation of the Wnt/β-catenin pathway and its strong tumor-promoting effects in EOC, it is highly possible that inhibition of the pathway will have strong therapeutic potentials. More clinical studies should be done in EOC to explore this possibility.

## Data Availability

N/A

## Abbreviations

ABCG2: ATP-binding cassette sub-family G member 2
AKT: RAC-alpha serine/threonine-protein kinase
ALDH: Aldehyde dehydrogenase
APC: Adenomatous polyposis coli
ATP: Adenosine triphosphate
bFGF: Basic fibroblast growth factors
BMI1: Polycomb complex protein BMI-1
BMP-10: Bone morphogenetic protein-10
BRCA: Breast cancer susceptibility protein
CBL: C asitas B-lineage Lymphoma
CBP: Creb-binding protein
CCC: Clear cell carcinoma
CCNG2: Cyclin G2
CCNY: Cyclin Y
CD: Cluster of differentiation
CDK: Cyclin-dependent kinase
circRNA: Circular RNA
CK1: Casein kinase 1
C-kit: Mast/stem cell growth factor receptor Kit
CSC: Cancer stem cell
CUL4A: Cullin-4a
DACT1: Dishevelled binding agonist of β-catenin 1 or Dapper 1
DKK: Dickkopf-related protein
DVL: Dishevelled
EC: Endometrioid carcinoma
ECM: Extracellular matrix
EMT: Epithelial-to-Mesenchymal Transition
EOC: Epithelial ovarian cancer
EPCAM: Epithelial cell adhesion molecule
FHL2: Four and half LIM domain protein 2
FILIP1L: Filamin A interacting protein 1-like
FN: Fibronectin
FOXA2: Forkhead box protein A2
FOXM1: Forkhead box protein M1
FOXO3: Forkhead box protein O3
FZD: Frizzled receptor
GSK3β: Glycogen synthase kinase 3β
hCTR1: Human copper transporter 1
HGSC: High-grade serous carcinoma
ICAT: β-catenin interacting protein 1
IDO: Indoleamine 2, 3-dioxygenase
IL: Interleukin
Jak: Janus kinase
JRK: Jerky protein homolog
KIAA0101: PCNA-associated factor
LGR: Leucine-rich repeat containing G protein-coupled receptor
LGSC: Low-grade serous carcinoma
lncRNA: Long non-coding RNA
LPA: Lysophosphatidic acid
LPAR: Lysophosphatidic acid receptor
LRP: Low-density lipoprotein receptor-related protein
MARCH7: Membrane-associated ring finger protein 7
MC: Mucinous carcinomas
miRNA: micro RNA
MMP: Matrix metalloproteinase
NANOG: Homeobox protein NANOG
OCT: Octamer-binding protein
PARP: Poly (ADP-ribose) polymerase
PD-L1: Programmed cell-death ligand
PI3K: Phosphoinositide 3-kinase
PIK3R3: Phosphatidylinositol 3-kinase regulatory subunit gamma
PP2A: Protein phosphatase 2
PYGO: Pygopus
Rab14: Ras-related protein Rab-14
Rnf43: Ring finger protein 43
RSPO: R-spondin
SCF: Stem cell growth factor
SCF-βTrCP: skp, cullin and f-box containing complex-β-transducin repeats-containing protein
SFRP: Secreted Frizzled-Related Protein
siRNA: Small interfering RNA
Skp1: S-phase kinase-associated protein 1
SNP: Single nucleotide polymorphism
SP1: Specificity protein 1
STAT: Signal transducers and activators of transcription
TCF/LEF: T cell factor/lymphoid enhancer factor
TCGA: The Cancer Genome Atlas
TET: Ten-eleven translocation methylcytosine dioxygenase
TF3: Theaflavin-3, 3'-digallate
TG2: Tissue transglutamase 2
TGFβ: Transforming growth factor beta
Th17: T helper 17 cell
TNKS: Tankyrase
Treg: Regulatory T cells
VEGF: Vascular endothelial growth factor
YY1: Ying Yang1
Znrf3: Zinc and ring finger 3
